# Direct visualization of virus removal process in hollow fiber membrane using an optical microscope

**DOI:** 10.1038/s41598-020-78637-z

**Published:** 2021-01-13

**Authors:** Miku Ayano, Yoshiyuki Sawamura, Tomoko Hongo-Hirasaki, Takayuki Nishizaka

**Affiliations:** 1 Department of Physics, Gakushuin University, 1-5-1 Mejiro, Toshima-ku, Tokyo, 171-8588 Japan; 2 Global Marketing Department, Bioprocess Division, Asahi Kasei Medical Co., Ltd., Chiyoda-ku, Tokyo, 101-8101 Japan

**Keywords:** Biomedical engineering, Biophysics

## Abstract

Virus removal filters developed for the decontamination of small viruses from biotherapeutic products are widely used in basic research and critical step for drug production due to their long-established quality and robust performance. A variety of imaging techniques have been employed to elucidate the mechanism(s) by which viruses are effectively captured by filter membranes, but they are limited to ‘static’ imaging. Here, we propose a novel method for detailed monitoring of ‘dynamic process’ of virus capture; specifically, direct examination of biomolecules during filtration under an ultra-stable optical microscope. Samples were fluorescently labeled and infused into a single hollow fiber membrane comprising cuprammonium regenerated-cellulose (Planova 20N). While proteins were able to pass through the membrane, virus-like particles (VLP) accumulated stably in a defined region of the membrane. After injecting the small amount of sample into the fiber membrane, the real-time process of trapping VLP in the membrane was quantified beyond the diffraction limit. The method presented here serves as a preliminary basis for determining optimum filtration conditions, and provides new insights into the structure of novel fiber membranes.

## Introduction

Removal of virus particles has long been a critical issue in production of human plasma derivatives and biotherapeutic agents. Planova series of regenerated cellulose fiber were launched by Asahi Kasei Inc. for this purpose in 1989, and have effectively been employed to remove virus-sized contaminants from final products with high reliability. Numerous advanced filters have subsequently been developed and used widely for over three decades, serving an important role in improving the safety of drug products^1,2^.

Planova filters share a common structure of single hollow fibers comprising a microporous membrane^3^. Contaminants larger than the size of a protein become trapped in the membrane according to a size-exclusion mechanism. However, if the membrane worked as a simple sieve, then dominant flow paths and fluid channels would instantly become clogged by large particles. A more detailed understanding of the filtration process is therefore required to facilitate the development of more functional and efficient virus removal filters. As a necessary first step, methods need to be developed to localize accurately virus particles captured in filter membranes. These methods would clarify reasons of various problems commonly encountered during filtration in actual production sites for medical drugs, such as clogging of filters with highly concentrated proteins.

Various visualization methods have been applied to clarify the mechanisms of virus particle capture using Planova filters. Firstly, the capture of gold particles was observed using transmission electron microscopy (TEM)^3^. Similar to viruses in size, these particles were used as a virus model. Their capture within the membrane was quantified based on particle number and a membrane thickness function. Changes in their distribution in response to the particle number burden were also analyzed as an increase in captured particle number, and a shift in the distribution peak toward the inside of the membrane wall. Consequently, a multi-step filtration mechanism was proposed, in which particles are captured in a pore network comprising repeated large pores (voids) connected by narrow pores (capillaries) within the membrane. Extending the aforementioned TEM approach confirmed that both parvovirus B19 (Ref.^4^) and porcine parvovirus^5^ particles, with the size of ~ 20 nm in diameter, can be captured using the hollow fiber membranes. These studies demonstrated that TEM visualization is well-suited to the precise measurement of particle number and size within membranes. Further, the capture of parvovirus B19 was achieved using two virus removal filters, Planova 15N and 20N, under actual process solutions containing proteins such as intravenous immunoglobulin G (IgG), albumin, antithrombin and haptoglobin^6^. Fluorescent antibody staining of parvovirus B19 enabled the visualization of virus localization within the membrane after filtration under permeate volume and protein solution conditions resembling actual manufacturing processes with parvovirus B19-spiked solutions.

These static visualization methods have clarified the final state of virus particles captured in the membrane. However, while static visualization enables the observation of virus capture post-filtration, the processes by which the virus particles reach the dense middle layer of the membrane where they are captured are still an enigma. Here, we propose a new method for visualizing and describing the dynamic processes associated with virus particle capture. We consider that this method will facilitate a deeper understanding of filtration phenomena and the optimization of filtration conditions.

## Results

### Accumulation of single hollow fiber under microscope

**Figure 1:**
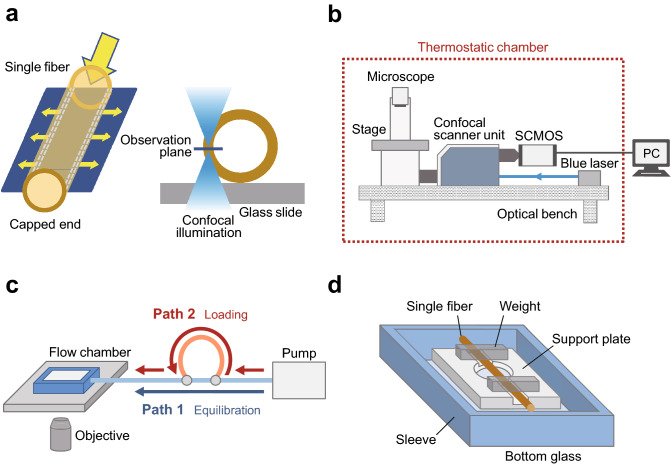
Experimental setup used to visualize biomolecules behavior in single virus removal hollow fiber membrane. (**a**) Schematic of the configuration and flow characteristics of medium through the hollow fiber membrane. (**b**) Diagram of the optical microscope and recording equipment setup. (**c**) Tubing diagram. (**d**) Sample observation chamber.

We attempted to visualize the behavior of virus particles inside a cuprammonium regenerated-cellulose hollow fiber membrane. To the best of our knowledge, we obtained real-time sequential images of virus accumulation in a hollow fiber membrane for the first time. A single hollow fiber, measuring ~ 0.5 mm in diameter as used in commercial Planova 20N filters, was prepared capping one end and then set in parallel to the sample plane of an inverted microscope (Fig. 1a, left). Confocal illumination was applied to obtain a sectioned image of the fiber membrane (Fig. 1a, right). Briefly, confocal illumination enables the detection of the sample only in the z-plane of the ‘confocal volume’, which minimizes signal from other samples located in other focal planes. This approach increased the reliability and reproducibility of the main signal with very low background signal. The microscope was equipped with a confocal scanner unit and hand-made optical elements for a blue laser. The experimental components required for the observations were set up on an optical bench and enclosed in a thermostatic chamber to minimize sample drifting^7^ (Fig. 1b). The open end of the fiber was connected to a syringe pump, by which the solution was injected into the fiber at a constant flow rate (Fig. 1a,left,c). We also set up a sample injector with two paths. After the inner pressure and position of the fiber was equilibrated by infusing the medium through Path 1, a sample was loaded through Path 2 by switching nodes (Fig. 1c). Using this simple setup, any biomolecule labeled with a fluorescent probe can be injected into a single hollow fiber.

To achieve accurate time-lapse recording, a 0.5 mm-thickness aluminum support plate was prepared to hold the fiber during observation. The hollow fiber was first gently guided into a 0.5 mm-deep recess in the plate and then held in position by two weights from the top (Fig. 1d). The support plate also had an aperture measuring 5 mm in diameter, through which the image of the membrane section was acquired. These modifications resolved the issues associated with fiber movement, enabling quantitative evaluation of sample signals from inside the membrane. As a result, displacement of the observation system in the *x*-, *y*- and *z*-planes was negligible and recording could be undertaken over several hours. The distance between the surface of the bottom glass and the objective lens was kept constant using a commercially available feedback system^7^ (see “Methods” for details). In all following experiments, hollow fibers were thus prepared and observed.

### Continuum injection of virus-like particles

We prepared a fluorescently labeled sample of virus-like particles^8,9^ (VLP) and investigated whether our observation system was sensitive enough to resolve biomolecule behavior of using an appropriate time resolution. VLP were labeled with an NHS ester-activated dye, DyLight 488, dialyzed, and then subjected to chromatography prior to infusion into the fiber (see “Methods” for details). The chromatography process was critical for successive measurements, because residual capsid proteins that do not form whole VLP are still fluorescent and thus need to be separated from the sample to ensure that only the behavior of whole virus particles are tracked.

**Figure 2:**
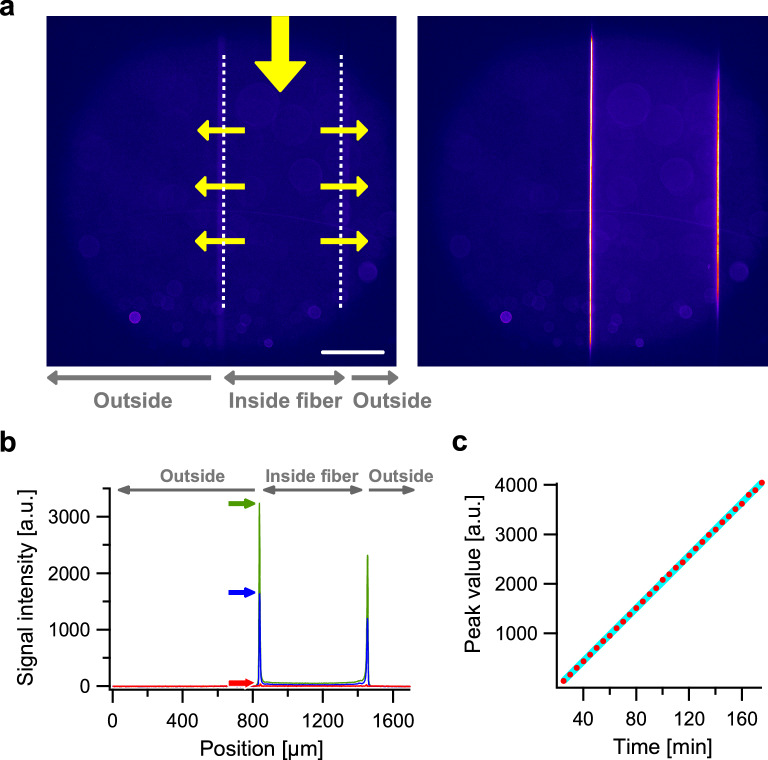
Visualization of the accumulation of virus-like particles (VLP) in the hollow fiber membrane using the continuum injection method. (**a**) Pseudo-color representation of fluorescent images taken by the confocal microscope. Scale bar, 300 µm. Left, whole view of a single hollow fiber membrane. White dotted lines indicate the positions of left and right edges of the lumen in the hollow fiber. Yellow arrows show the generalized movement of the solution, which was infused from the top in this figure, and then flowed sideways through the membrane. Right, image at 175 min after the VLP reached the observation area. (**b**) Signal intensity profile along the axis perpendicular to the hollow fiber. Red, blue and green lines are profiles recorded at 0, 60 and 120 min, respectively. Arrows indicate peak intensities. (**c**) Time course of the retention of the VLP trapped in the membrane as left peak values of profiles (arrows in **b**). Cyan line shows a linear regression.

To visualize the process of virus removal, the buffer solution was first infused into the hollow fiber for a few hours to equilibrate the observation system; specifically, regarding conditions such as fiber moisture, lumen pressure, and sample drift. A section of the fiber membrane was then captured as a bright-field image and as a dim fluorescent image with autofluorescence (Fig. 2a, left). The lumen diameter of the fiber was ~ 600 µm in our measurement condition (see “Methods” for details). ‘Continuum injection’ was then performed in which fluorescent VLP were continuously infused into the lumen using the injector equipped with the sample tube (Fig. 1c). Notably, when the front fraction of VLP reached the observation area, a clear signal appeared at defined layer inside the membrane. Using our ultra-stable observation setup (see “Methods” for details; Fig. 1d), we detected the signal at the same position in the membrane for a few hours (Fig. 2a, right) without adjusting either the sample stage or the objective lens. Parameters such as exposure time, acquisition interval, and power of the laser were chosen in order to determine the optimum conditions under which the fluorescent signal from the trapped VLP increased over time (Fig. 2b,c; Supplementary Movies 1 and 2). To our knowledge, this is the first report to visualize virus accumulation inside a hollow membrane filter in real-time.

The signal intensity profile of the hollow fiber membrane showed two peaks due to intra-membrane accumulation of fluorescent VLP, as shown in Fig. 2b in which *x*-axis is perpendicular to the fiber axis. Because the intensity of a single beam of a laser exhibits 2-D Gaussian profile, ununiformity of excitation illumination is unavoidable. We aligned the illumination peak to the center of the field view and set the left edge of the fiber section there, and so the left peak was brighter than the right one. For quantitative analysis, we took values from the left peak, and plotted the change in peak values over time as shown in Fig. 2c. The finding showed that peak values increased over time (straight cyan line in Fig. 2c), implying that the experimental setup was robust; specifically that the positional feed back regarding objective, flow rate, and linearity of the detection sensor were all optimal and functioning normally. Furthermore, the observed linearity suggests that the membrane was not saturate by this range of viral retention; specifically, up to 4000 a.u. with an exposure time of 5 s and a laser power of 3 mW mm^−2^ in the sample plane.

### Shot injection of protein

**Figure 3:**
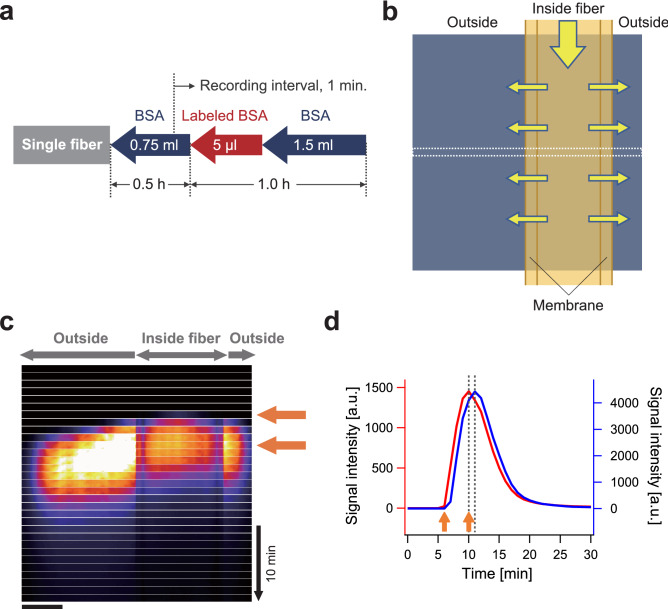
Behavior of a model protein passing through the membrane. (**a**) Diagram showing shot injection of the fluorescently labeled BSA. After equilibration of the solution containing 10 mg mL^−1^ of unlabeled BSA, 5 µL of BSA labeled with 20 µM fluorophore was infused as a single shot, and subsequently unlabeled BSA were infused. (**b**) Schematic diagram of the observation in a single image. (**c**) Image sequence comprising narrow bands aligned in the *y*-axis. Images were taken at 1-min intervals. The top arrow shows the frame when fluorescence started to appear inside the fiber. The bottom arrow shows the moment of peak signal intensity inside the membrane. Scale bar, 300 µm. (**d**) Time course of the change in intensity inside (red) and outside (blue) the membrane. Two dotted lines represent the times of peak signal intensity.

To examine the difference of behavior between a particle with the size of virus and a protein, a model protein (bovine serum albumin, BSA) was fluorescently labeled and visualized. In addition, we applied a ‘shot injection’ method in which a certain amount of fluorescent BSA was shot into the flow path after equilibration with unlabeled BSA (Fig. 3a). Images were recorded at 1-min intervals, from just before the sample reached the fiber membrane. We expected that we would be able to detect the moments when the BSA reached the fiber membrane and disappeared using this procedure.

To represent the change in signal intensity at once, a narrow band (the square region indicated by white dotted line in Fig. 3b) was taken from a single picture in the image sequence, and the bands were aligned in the *y*-direction (Fig. 3c). When the leading fraction of the sample reached this observation area, the center part of the hollow fiber became bright (top arrow in Fig. 3c). As the area inside the fiber membrane became brighter, the outer edge of the membrane also became brighter due to the amount of light distribution, despite retention of the emitter inside the membrane. Subsequently, however, fluorescent BSA passed through the membrane and started to appear in the region outside the fiber. Notably, the distribution of brightness gradually dispersed outside the membrane, indicating that the fluorescent protein passed through the membrane quickly and unidirectionally. The uniform flow resulted in the intensity peak being delayed in the area outside the membrane (dotted lines in Fig. 3d). Finally, no signal was observed inside the hollow fiber, as most of the fluorescent BSA had been washed out of the observation area. These findings contrasted markedly with those of the VLP experiment, in which the VLP, with the size of ~ 20 nm (Ref.^9^), were accumulated in the membrane producing the sharp peak as observed in Fig. 2b. Comparison of the results obtained from the continuum injection of VLP and shot injection of protein experiments clearly showed that biomolecules that are similar in size to typical target proteins, ~ 5 nm, can efficiently pass through Planova 20N filter hollow fiber membranes, while particles with the size of viruses are trapped.

### Shot injection of VLP and super-resolution analysis of VLP position in the membrane

**Figure 4:**
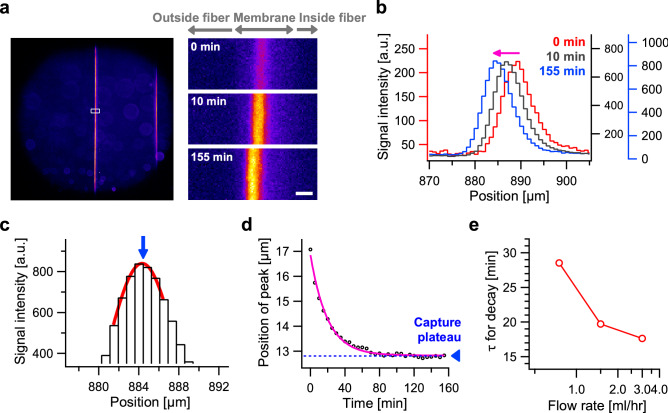
Real-time image of VLP capture in the membrane visualized using the shot injection method. A total of 200 µL of fluorescently labeled VLP were applied in a solution containing 10 mg mL^−1^ of h-IgG to reproduce a standard virus-filtration process. (**a**) Left, confocal image at 155 min. Right, magnified view of white box in (**a**) at 0, 10 and 155 min. Scale bar, 10 µm. (**b**) Shift in intensity profile at 0 (red), 10 (black) and 155 min (blue). (**c**) Magnified profile at 155 min to show localization of the peak with a sub-pixel resolution. (**d**) Time course of the peak intensity profile of VLP. (**e**) Flow rate dependency of the time constant of the single-exponential displacement.

In the measurements shown in Fig. 2, the fluorescent VLP were continuously infused into a single hollow fiber where they accumulated over time (cf., Fig. 2c). Here, ‘shot injection method’ was used to infuse the VLP sample in order to visualize the progression of VLP accumulation in the filter membrane at a high level of temporal resolution (Fig. 4). We used the same microscope system that was employed for the shot injection of protein experiment described above (Figs. 1b–d, 3a). Briefly, the buffer containing IgG (h-IgG) was used in a series of observations to mimic actual virus removal process from protein specimen; 200 µL of fluorescent VLP were injected into a hollow fiber membrane at a flow rate of 1.5 mL h^−1^. First, the moment when the leading fraction of VLP reached the inner edge of the membrane was clearly distinguished (time point ‘0 min’ in Supplementary Movie 3 and Fig. 4a, right top). Note that the inner most part of the membrane could not be decisively indicated because the boundary between inner surface and the lumen was blur even under phase-contrast microscopy, and so we could not measure the peak position from the inner surface. The signal intensity value subsequently increased before remaining nearly constant (Supplementary Movie 3 and Fig. 4a, right, top to bottom), indicating all of the injected sample was successfully trapped in the membrane. Unexpectedly, the position of the trapped VLP shifted ~ 4 µm outward with time, as is clearly shown in sequential micrographs (Fig. 4a, right) and intensity profiles (Fig. 4b).

To quantitatively analyze the capture process more accurately, we extracted the detailed peak positions from pixelized data points using an appropriate function; in this case, we used a one-dimensional Gaussian (red curve in Fig. 4c; blue arrow indicates the peak). This analytical procedure is analogous to the extremely precise localization of biomolecules previously demonstrated using a highly sensitive camera and two-dimensional image data^10^, as well as super-resolution microscopic techniques such as PALM and STORM^11,12^. Using our microscope setup, the projection of the magnified image onto the camera sensor was 830 nm per one pixel, but the precision of localization of the VLP peak was typically at the sub-10 nm scale in the above analysis (see “Methods” for details). The VLP movement within the membrane could therefore be quantified. As clearly shown in the time course data of the peak position (Fig. 4d), which was obtained by both real-time membrane recording (Supplementary Movie 4) and super-resolution analysis (Fig. 4a–c), VLP were displaced in the membrane in a single-exponential manner (red line in Fig. 4d) and paused at a defined location in the membrane after 2 h (blue arrowhead in Fig. 4d). Although this observation indicates that the middle structure of the membrane is sufficiently dense to trap viruses under conditions of constant flow of the medium, the displacement behavior cannot be explained by a simple molecular-sieve model, in which particles with a defined homogeneous size are stopped immediately, and with an unmeasurably short dwelling time, by the dense pore structure. Additionally, the ‘time constant’ required to reach these plateaux decreased as the flow rate increased (Fig. 4e). These observations prompted consideration of the property of drag of the membrane and how this is imposed on VLP in Supplementary Discussion.

## Discussion

We developed an advanced experimental setup that allows for the real-time visualization over several hours of any biomolecule inside single hollow fiber membrane for virus removal. Three key techniques were employed to set up the system: confocal optics for sectioning sequential images (Fig. 1a), an ultra-stable microscope equipped with a custom-made sample stage and support plate (Fig. 1d) for use in a thermostatic chamber^7^, and high-spec syringe pump with a injector (Fig. 1b,c). The support plate was critical for fixing single hollow fibers, which are too soft and flexible to be held stably in a conventional observation setup. This novel approach will be helpful for assessing membrane filters with different polymer architectures. It may also be applicable to other fundamental and general polymer tools in the chemical-engineering research field, such as chromatography purification, hemodialysis membrane, or gel filtration, in the near future.

To the best of our knowledge, these results represent the first sequential images of virus entrapment inside the polymer macromolecule of a hollow fiber membrane in real time. Unlike the VLPs, which were accumulated at a specific region in the membrane (Fig. 2), a model protein, BSA, passed through the membrane (Fig. 3). Nazem-Bokaee et al. produced confocal images of polystyrene beads in Planova filter membranes^13^; however, their images were all snapshots without any well-defined single peaks in the signal intensity profile. Leisi et al. examined the virus retention profile in a planar membrane using laser scanning microscopy; however, their fluorescent images were taken only after filtration^14^. In contrast, our observations directly demonstrated the functionality of the virus removal membrane filter, in this case Planova 20N, in a way that could be visualized and recorded in real time. In addition, we quantitatively demonstrated movement of a single well-defined fluorescent peak associated with a biomolecule specimen (Fig. 4b).

Finally, by using super-resolution analysis over the diffraction limit, VLP movement in the virus capture process was precisely quantified in a single-exponential manner (Fig. 4d). Because we observed the movement of the peak of the intensity profile, one may argue that each particle is trapped and immobilized in a certain position due to the saturation of voids, and other particles passed early-retained VLP. To address this point, we plotted the subtraction profile from two curves with an interval of doubled time constant, i.e., the intensity profile at 10-min was subtracted from that at 50-min. Importantly, negative values were apparent in the curve (Supplementary Figure 1). This implies that the assemblage of injected VLP traveled as a mass in the membrane over the timescale used in this study. The VLP behavior accompanying single-exponential motion could provide clues as to how the filter membrane captures virus-sized particles. One simple explanation is that the membrane structure has a high resistance load as the flow forces particles deeper into the membrane. Since two forces—the flow force and membrane resistance *F*_m_—are always balanced in a system with a low Reynolds number, the equation *F*_m_(*x*) + η × (*v*_s _– *v*) = 0 can be obtained as a first approximation; where, η is the drag coefficient of the fluid against the particle, *v*_s_ is the flow velocity and *v* is the particle velocity. To reproduce our observation with single-exponential movement to reach the final trapped position, the function of *F*_m_(*x*) in the equation should be in the form of – *k* × *x*, where *k* is the parameter that acts as the spring constant (see Supplementary Discussion for details). This idea also explains why the VLP were captured with a different time-constant depending on the flow rate as observed in Fig. 4e. While the above equation is based on several assumptions, it represents the first attempt at quantitatively evaluating real-time particle behavior in an artificial polymer macromolecule. Applying this equation to other theories related to membrane micro-structures, such as capillary and void compositions^3^, is the next great challenge.

Applying the calibration factor calibrated in our measurements, 10^–17^ mol s a.u.^–1^ (see Supplementary Analysis for details), the number of VLP that were trapped in a single fiber membrane over several hours (Fig. 2c) was estimated to be the order of 10^8^ particle, using the following calculation: (4000 a.u.)/(5 s) × (10^–17^ mol s a.u.^–1^) × (6.0 × 10^23^ mol^–1^) ~ 5 × 10^9^ fluorophore ~ 1 × 10^8^ particle, assuming that one particle comprises 60 capsid proteins with 100% labeling efficiency as VLP size was similar to infectious MVM^9^. For the size of the single fiber membrane that we used, the density of retained particles was calculated to be the order of 10^12^ particle m^–2^, using the following calculation: (10^8^ particle)/(9 × 10^–5^ m^2^) ~ 1 × 10^12^ particle m^–2^. Lower than previous estimates obtained using an electron microscope in a virus-spiking experiment^5^, this value would be helpful to ensure the safety and efficacy of the virus removal process for general users. To understand the filtration process in greater detail at a microscopic scale, the next step in virus particle visualization would be single-virus tracking, as achieved in a series of single molecule biophysics studies^15,16^. Interestingly, the number of actual viruses visualized per pixel in the red curve shown in Fig. 4b is estimated to be only one particle in single camera pixel, using the following calculation: (200 a.u.)/(4000 a.u.) × (1 × 10^8^ particle)/(9 × 10^–5^ m^2^) × (0.83 µm) × (14.5 µm) ~ 1 particle, based on the premise that the capture area is (pixel size) × (confocal depth) = (0.83 µm) × (14.5 µm). Thus, by observing an extremely diluted VLP sample at an appropriate recording rate while maintaining a similar s/n ratio, the signal from a single VLP could, in principle, be detected with one pixel. It is therefore expected that the behavior of viruses in artificial membranes will be realized at the single-particle level in the very near future.

## Materials and methods

### Preparation of samples

#### VLP

Non-infectious virus-like particles (VLP) are comprised of the capsid proteins of minute virus of mice (MVM) and mimic the physicochemical properties of live infectious viruses. In this study, VLPs (Cygnus Technologies, LLC) were labeled with a reactive fluorescent agent (DyLight 488 NHS-Ester ;Thermo Fisher Scientific) for 1 h at R.T. in 50 mM NaCl, 1 mM EDTA and 20 mM Tris at pH 7.5. The labeled VLPs were dialyzed using a dialysis membrane tube (Spectra/Por 4; Spectrum Laboratories) to remove any unreactive agent. Briefly, 1 mL sample was enclosed in dialysis tube against 400 mL fluid for 3–4 h. The external fluid was exchanged four times. After the addition of 0.5 mM glycine to terminate the reaction, the labeled VLPs were stored in the presence of 1.5 mM NaN_3_ at 4 °C. The labeled VLPs were further purified before observation, by high-pressure liquid chromatography (HPLC; ÄKTAexplorer 10S; GE Healthcare), using a pre-packed column (HiPrep 16/60 Sephacryl S-500HR; GE Healthcare) in DPBS buffer (2.6 mM KCl, 140 mM NaCl, 1.5 mM KH_2_PO_4_ and 8 mM Na_2_HPO_4_ at pH 7.5).

#### BSA

Commercial bovine serum albumin (BSA; Sigma Aldrich) was first purified by HPLC with a repacked column (Superdex 200 10/300GL; GE Healthcare), and fluorescently labeled according to the above procedure for preparing VLPs; however, some of the buffer conditions were changed (0.15 M NaCl and 0.1 M sodium phosphate at pH 7.2–7.5). Labeled BSA was further purified with the same column before observations were performed.

#### H-IgG

Medical-grade human IgG extracted from donated blood (Venoglobulin IH; Japan Blood Products Organization) was used in experiments in Fig. 4 in order to reproduce a standard protocol for virus removal to purify target proteins. The h-IgG buffer was replaced with DPBS using a desalting column (Sephadex G-25 in PD-10; GE Healthcare) before observations were performed.

### Flow chamber and pump

The custom-made polycarbonate sleeve measured 70 × 50 × 15 mm and had a square hole at the center. A glass slide (No. 1, 0.12–0.17 mm thickness, 70 × 50 mm; Matsunami Glass Ind., Ltd.) was attached to the bottom of the sleeve such that the whole construction functioned like a flow chamber (Fig. 1d). A custom-made aluminum support-plate with a thickness of 0.5 mm was set at the bottom of the chamber, into which ~ 5 mL DPBS was infused. A single Planova 20N hollow fiber (Asahi Kasei Medical Co. Ltd.), in which the inner surface area was calculated to be 9.4 × 10^–5^ m^2^, was immersed in DPBS, connected to a PTFE tube (Flon Industry Co. Ltd.) that had been filled with DPBS in advance, and then gently guided into a 0.4 mm-deep recess in the aluminum support-plate. The fiber was held in position by two weights on the top, and observed through a 5-mm hole in the plate. Media and samples were infused into the PTFE tube using a high-pressure valve (MX Series II MXP9900-000; IDEX Health & Science) equipped with a syringe pump (Legato 210; KD Scientific) and three different sample loops of 5 µL, 200 µL and 5 mL in volume (PEEK; IDEX Health and Science) were used for the experiments shown in Figs. 2, 3, and 4 respectively. Inner pressure was measured using a digital pressure gauge (QuickStart Pressure Sensor; IDEX Health and Science). A buffer flow rate of 1.5 mL h^−1^ produced a pressure of ~ 40 kPa in the tubing system.

### Optical microscopy, camera, imaging procedure and setup

The fluorescent signal inside the fiber membrane was visualized with a confocal scanner unit (CSU-W1; Yokogawa) and CMOS camera (Zyla; Andor) under an inverted microscope (Ti-E; Nikon) equipped with 10 × objective lens (S Flour, N.A. 0.50; Nikon), LED (pE-100 660 nm; CoolLED), on an optical bench (RS2000; Newport). The beam of a blue laser (OBIS 488; Coherent) was introduced to the confocal scanner unit using a configuration of optical components, such as mirrors, lenses, a mechanical shutter mounted on the optical bench. The ultra-stable customized sample stage (Chukousha) was controlled by three actuators (SGSP-13ACTR; Sigma Koki). Most of the experimental equipment, except the computer equipment, were enclosed in a custom-made thermostatic chamber (Nihon Freezer), and all operations were performed from outside the chamber. Measurements were conducted at 23 ± 0.2 °C. The camera, laser, and filter wheel in the microscope were all operated by a single software program (IQ3; Andor). To acquire the sequential time-lapse images, the exposure times and intervals between snapshots were set to 5.0 s and 5 min for the images shown in Figs. 2 and 4, and 0.3 s and 1 min for that in Fig. 3, respectively. Bright-field images corresponding to every fluorescent image were also taken to monitor the condition and position of the hollow fiber membrane. The signal intensity profile in each frame was analyzed using custom-made macros running on the data-analysis software (Igor Pro 7; WaveMetrics, Inc.). In the analysis for experiment in Fig. 4, a two-dimensional image was once converted to a one-dimensional sequence by averaging 150 pixel-values in the same longitudinal axis as the hollow fiber membrane. The precise position beyond the diffraction limit was determined by applying a one-dimensional Gaussian function to the above sequence. To compensate any drift or slight expansion of the membrane, the peak position of VLP distribution was measured from the outer edge position, which was localized from the bright-field image taken after each acquisition frame, and plotted in Fig. 4d.

## Supplementary Information

Supplementary Information.

Supplementary Video 1.

Supplementary Video 2.

Supplementary Video 3.

Supplementary Video 4.
