# Supplementary material for: Direct visualization of virus removal process in hollow fiber membrane using an optical microscope
Source: Sci Rep. 2021 Jan 13;11:1095. doi: 10.1038/s41598-020-78637-z (PMC7806983; doi:10.1038/s41598-020-78637-z)
Supplement: Supplementary file 1 — Supplementary Information. [file 41598_2020_78637_MOESM1_ESM.pdf]

Direct Visualization of Virus Removal Process in Hollow Fiber Membrane Using an Optical Microscope

Miku Ayano[1], Yoshiyuki Sawamura[2*], Tomoko Hongo-Hirasaki[2], Takayuki Nishizaka[1]*

[1]Department of Physics, Gakushuin University, 1-5-1 Mejiro, Toshima-ku, Tokyo 171-8588, Japan

[2]Global Marketing Department, Bioprocess Division, Asahi Kasei Medical Co., Ltd., Chiyoda-ku, Tokyo 101-8101, Japan

*Requests for materials and correspondence should be addressed to
Y.S. (sawamura.yd@om.asahi-kasei.co.jp)
or T.N. (takayuki.nishizaka@gakushuin.ac.jp; Tel.: (+81)-3-5904-9372).

Supplementary Figure 1, 2

Supplementary Analysis

Supplementary Discussion

Supplementary Movies 1, 2, 3, & 4

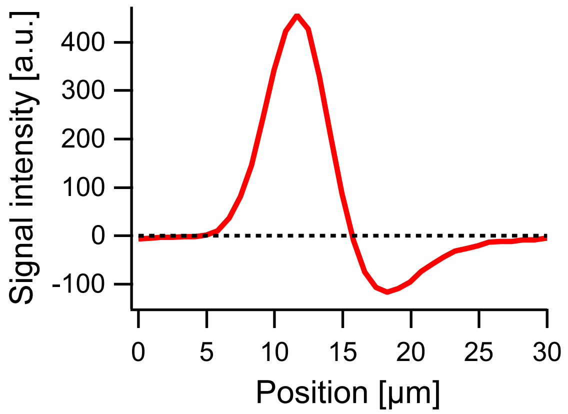

Supplementary Figure 1. The subtraction profile. The original intensity profile at 10-min was subtracted from that at 50-min. Points showing negative values around 18-μm directly indicate that VLP traveled as a mass in the membrane in a single-exponential manner with a time constant, τ, of ~20 min (see Fig. 4d in the main manuscript). Profiles at 50-min and 10-min were chosen as the time difference between two plots were ~2τ.

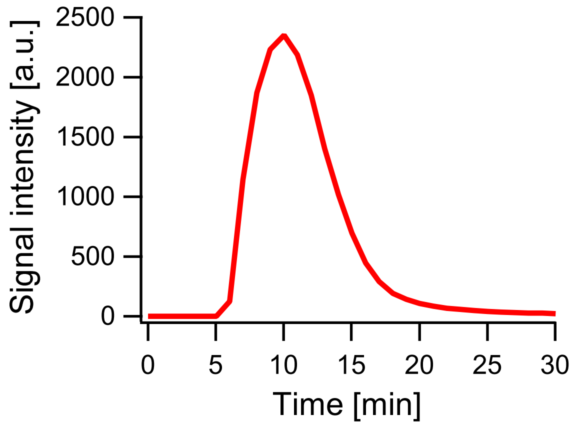

Supplementary Figure. 2. Time course of the changes in membrane signal intensity after a ‘shot injection’ of fluorescently labeled BSA. The signal intensity of pixels corresponding to the inside of the membrane was plotted.

Supplementary Analysis

From the data presented in Figs. 3 and S1, we estimated the calibration factor, i.e., mol per unit fluorescent value (a.u.) measured in our observation condition with the 1-s exposure time.

In our measurements, 5 μL fluorescently labeled BSA, to which 23 μM DyLight488 was coupled, was infused into the hollow fiber membrane. Thus, a total of (5 μL) × (23 μM) ~ 10^{-10} mol of fluorophore passed through the membrane in all directions. We detected this process by observing one representative pixel and plotted the time course of changes in signal intensity in Fig. S2. From the integrated value of the curve shown in Fig. S2, the total amount of fluorescent signal was estimated to be 1.6×10^{5} a.u. We assume that all of the molecules that we injected were detected in less than 300 ms of exposure time because the curve in Fig. S2 is contiguous. The expected total fluorescent signal was corrected to be (1.6×10^{5} a.u.) × (60 s / 0.3 s) ~ 3.1×10^{6} a.u., as the time interval between exposures was 60 s. Based on these values, we estimated that the calibration factor was (10^{-10} mol) / [(3.1×10^{6} a.u.) / (0.3 s)] ~ 10^{-17} mol s a.u.$^{-1}$.

The dimensions of the single hollow fiber that we used had an inner diameter of ~600 μm and a length of ~5 cm. Therefore, the area of the inner wall of the membrane was estimated to be $\pi\times(600\times10^{-6}\ \mathrm{m})\times(5\times10^{-2}\ \mathrm{m}) \sim 9\times10^{-5}\ \mathrm{m}^2$.

Supplementary Discussion

In Fig. 4d in the main manuscript, we observed the process of virus-like particles (VLP) capture in a single exponential manner. Here, we discuss a possible scenario to explain our observation of VLP movement with an exponential function reaching a plateau.

We assume that there is a resistance force from the membrane (F_m) that acts against each particle, and that this force depends on the particle position (x) due to the dense structure of the membrane layers. As a simple model, fluid force (F_f), a function of the relative speed of the flow against the particle (v_r), is also applied to the particle. v_r is expressed as $v_s – v$, where v_s and v are the speeds of the fluid and particle, respectively. The above two forces are always balanced because particle inertia is neglected in a system with an extremely low Reynolds number, giving us the following equation as a first approximation.

$$F_m(x) + F_f(v_r) = 0$$

Suppose that F_f is proportional to the speed of the particle, as in the case of a simple Newtonian fluid, then the equation can be rewritten as:

$$F_m(x) = –F_f(v_r) = –\gamma \times v_r = –\gamma \times (v_s–v)$$

or

$$v = \gamma^{-1} \times F_m(x) + v_s \qquad (1)$$

where, γ is the drag coefficient of the fluid against the particle.

In an actual observation, the red curve in Fig. 4d is described as:

$$x = r \times \{1–\exp[–(t–t_0) \times \tau^{-1}]\} + x_0 \qquad (2)$$

where, t_0 is the moment when the fluorescent signal is detected for the first time at the position close to the inner edge of the membrane; x_0 is the peak position of the signal at $t = t_0$; τ is the time constant required to reach the position where the particle is captured and finally immobilized; r is a parameter indicating the depth that VLP can move in the membrane to reach the capture plateau. In Fig. 4d, we took the position of the outer edge of the membrane as $x = 0$, and thus the thickness of the membrane is nearly equal to x_0.

Also, in the coordinate of Fig. 4d, the fluid flow and the direction of the moving particle are negative, and so $r < 0$. Finally, equation (2) suffices the following differential equation as a solution:

$$dx/dt = v = -\tau^{-1} \times (x - x_0) + r \times \tau^{-1} \qquad (3)$$

To fulfill the equation (2) as a solution of (1) as well, (1) has to be substantially identical to (3). Therefore, by comparing the first terms of (1) and (3), we conclude that the function of $F_m(x)$ should take the form of $-k \times (x - x_0)$, which is a simple spring-like function having the potential energy of $E = 1/2\ k \times (x - x_0)^2$; where k is a parameter used as the spring constant. Additionally, we also get $\tau = r \times v_s^{-1}$ by comparing the second terms of (1) and (3). The result shown in Fig. 4e, in which the time constant decreased as the flow rate increased, roughly confirms the above relationship and thus supports the framework of our assumption discussed here.

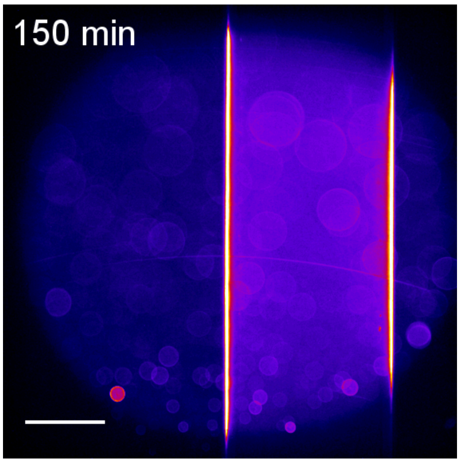

Supplementary Movie 1. Real-time virus removal filtration process. A single Planova™ hollow fiber membrane is embedded parallel to the glass and visualized from the bottom by a confocal microscope as a section. The thickness of the inner lumen is approx. 600 μm. Video starts from the frame representing the time point '–10 min', i.e., 10 min before the leading fraction of the sample reaches the observation area. Scale bar, 300 μm.

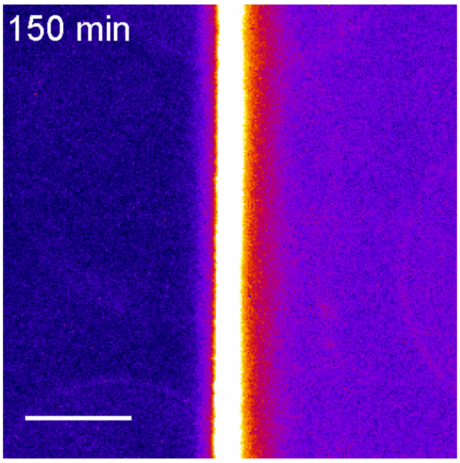

Supplementary Movie 2. Real-time virus removal filtration process. Magnified view of Supplementary Movie S1. Scale bar, 50 μm.

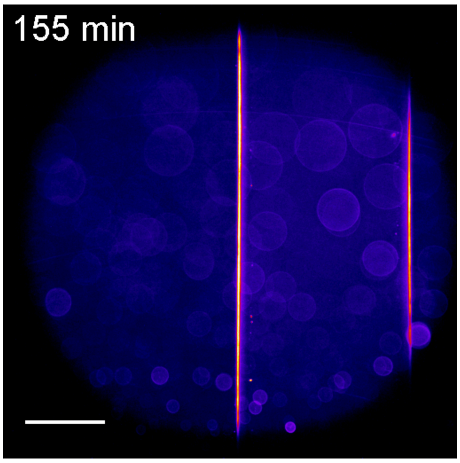

Supplementary Movie 3. ‘Shot injection’ method applied to fluorescent VLP. The single hollow fiber membrane was equilibrated with medium containing human IgG after which fluorescent VLP were injected. At time 0 min, the leading fraction of VLP reached the observation area. Scale bar, 300 μm.

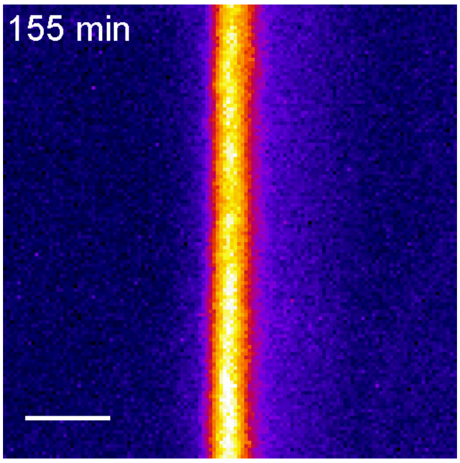

Supplementary Movie 4. ‘Shot injection’ method applied to fluorescent VLP. Magnified view of Supplementary Movie S3. Scale bar, 20 μm.
